# Prevalence of *Candida auris* in Canadian acute care hospitals among at-risk patients, 2018

**DOI:** 10.1186/s13756-020-00752-3

**Published:** 2020-06-10

**Authors:** Hector Felipe Garcia-Jeldes, Robyn Mitchell, Allison McGeer, Wallis Rudnick, Kanchana Amaratunga, Snigdha Vallabhaneni, Shawn R. Lockhart, CNISP C. auris Interest Group, Ghada Al-Rawahi, Elizabeth Brodkin, Marthe Charles, Jeannette Comeau, Ian Davis, Johan Delport, Tanis C. Dingle, Philippe Dufresne, Chelsey Ellis, Joanne Embree, Charles Frenette, George Golding, Linda M. Hoang, Susy Hota, Kevin C. Katz, Pamela Kibsey, Julianne V. Kus, Joanne M. Langley, Bonita E. Lee, Marie-Astrid Lefebvre, Yves Longtin, Kathy Malejczyk, Shazia Masud, Dominik Mertz, Michael R. Mulvey, Susan Poutanen, Dale Purych, Rajni Rantelidis, David Richardson, Ilan S. Schwartz, Stephanie Smith, Maxime-Antoine Tremblay, Titus Wong, Deborah Yamamura, Amrita Bharat

**Affiliations:** 1 CHU de Québec-Université Laval, Québec, QC Canada; 2 Public Health Agency of Canada, Ottawa, ON Canada; 3 Mount Sinai Hospital, Toronto, ON Canada; 4 US Centers for Disease Control and Prevention, Atlanta, GA USA; 5 National Microbiology Laboratory, Public Health Agency of Canada, H5050 - 1015 Arlington St, Winnipeg, MB R3E 3R2 Canada

**Keywords:** Candida, Cross infection, Drug resistance, Risk factors

## Abstract

To identify the prevalence of *C. auris* in Canadian patients who are potentially at risk for colonization, we screened 488 patients who were either hospitalized abroad, had a carbapenemase-producing organism (CPO), or were in units with high antifungal use. Two patients were colonized with *C. auris;* both had received healthcare in India and had a CPO. Among 35 patients who had recently received healthcare in the Indian subcontinent and were CPO colonized or infected, the prevalence of *C. auris* was 5.7%.

## Background

*Candida auris* is an emerging multidrug resistant pathogen associated with global hospital outbreaks [1]. Similar to other *Candida* species, the crude mortality rate of candidemia due to *C. auris* is 30–60% [2].

*C. auris* infection was first reported from Japan in 2009. Retrospective review of a large isolate collection identified an isolate from 1996, however, it appears that *C. auris* has very rarely caused human infection in the past [1]. Over the last decade, four distinct clades of *C. auris* have emerged and evolved independently, with frequent inter-hospital and inter-country transmission [2–5]. A single isolate belonging to a potential fifth clade was recently identified in Iran [6]. Whether the ongoing emergence of *C. auris* in different parts of the world will be mainly driven by the transmission of known clades, or whether new clades will continue to emerge remains uncertain.

There have been case reports of importation of *C. auris* by patients with recent exposure to healthcare in a country where *C. auris* is documented; these patients are often co-colonized with a carbapenemase producing organism (CPO) [7–10]. *C. auris* infection is often associated with underlying illnesses and outbreaks have been reported in intensive care units in multiple countries [11–13]. *C. auris* infection is also associated with previous exposure to antibiotics or antifungals [14, 15]. Immunocompromised patient populations such as those in haematology/oncology wards or solid organ transplant wards often receive prophylactic antifungals [16, 17]; additionally, antifungal use was reported to be higher in ICUs compared to non-ICU wards [18]. Many of the risk factors for *C. auris* colonization are also risk factors for CPO colonization; these common risk factors include critical illness and comorbid conditions, prolonged hospitalization, and receipt of antimicrobials [19]. This may explain why patients who are colonized with *C. auris* are often co-colonized with a CPO.

As of March 2020, 24 cases of *C. auris* colonization and infection in Canada have been voluntarily reported to the Public Health Agency of Canada. The first case of multi-drug resistant *C. auris* was identified in Canada in 2017 in a patient who had recently received healthcare in the Indian subcontinent and was co-colonized with a carbapenemase-producing organism (CPO) [20]. *C. auris* is reportable in only one of 13 Canadian provinces and territories, and there is little data or recommendations to inform screening programs. We aimed to identify the prevalence of *C. auris* in Canada to inform national guidelines for screening and infection prevention and control.

## Methods

The study included 23 acute care hospitals, of which 16 hospitals were participating in the Canadian Nosocomial Infection Surveillance Program (CNISP) and seven were Canadian Hospital Epidemiology Committee (CHEC) hospitals. We also included CPO colonized patients who were part of a prospective cohort study recruited from a 25 hospital research network, hereafter referred to as the “prospective cohort study” [21]. From September 4–November 6, 2018, hospitals screened patients in the following risk groups deemed by the study investigators to be potentially at risk for *C. auris* colonization: patients being admitted to study hospitals with recent hospitalization outside of Canada [Group 1]; patients being admitted to study hospitals with recent travel to the Indian subcontinent without hospitalization [Group 2]; CPO colonized or infected inpatients or outpatients [Group 3]; inpatients in hospital units that are associated with intensive antifungal use [Group 4]; and hospital contacts of a *C. auris* index case [Group 5] (Table 1). Patients who met more than one criteria were assigned to the first risk group identified. Patients in Groups 1 and 2 were identified through risk-based screening questions already in place in the hospitals. Patients in Group 3 included CPO-colonized inpatients and outpatients in the 23 participating hospitals as well as consenting participants in the other prospective cohort study of CPO colonized patients who had visits in September or October of 2018. Group 4 patients were identified by hospital census on a single day during the study period in each participating hospital. Group 5 patients were identified through active surveillance in hospitals with *C. auris* cases. The time periods defining recent travel (1 or 2 years) and overseas hospitalization (0.5, 1, or 2 years) varied as per each hospital policy. Data were collected using a standardized data collection form that included demographic and potential *C. auris* risk factors.

**Table 1:** Eligibility criteria for patient risk groups that were screened for *C. auris*

Group	Description	Notes
Group 1 (*n* = 92)	recent hospitalization outside of Canada	time periods defining recent overseas hospitalization (0.5, 1, or 2 years) varied as per each hospital policy
Group 2 (*n* = 117)	recent travel to the Indian subcontinent without hospitalization	time periods defining recent travel (1 or 2 years) varied as per each hospital policy
Group 3 (*n* = 104)	CPO colonized or infected inpatients or outpatients and consenting participants in a different prospective CPO cohort study	
Group 4 (*n* = 282)	units that are associated with intensive antifungal use	patients identified by hospital census on a single day
Group 5 (*n* = 0)	hospital contacts of a *C. auris* index case	screening as per each hospital policy

Two Eswabs™ (Copan Diagnostics, Murietta, CA) were held together to obtain combined bilateral axilla/groin swabs. One tube was inoculated directly onto chromogenic agar, either CandiSelect® (Bio-rad, 16 healthcare sites), Brilliance Candida® (Thermo Scientific, 6 sites), or other non-selective media (1 site). The second tube was shipped to the US Centers for Disease Control and Prevention (CDC) for culture with broth pre-enrichment and chromogenic agar as previously described [22]. Broth pre-enrichment allows for preferential growth of *C. auris* over other *Candida* species by employing high salinity (10% wt/vol NaCl), dulcitol as a carbon source, and incubation at elevated temperature (40 °C). *Candida albicans* colonies appear pink on CandiSelect® or green on Brilliance Candida® chromogenic agar. *C. auris* was identified by screening non-*albicans* colonies identified on culture by MALDI-TOF (Bruker Biotyper or Vitek MS). Isolates underwent whole genome sequencing on the NextSeq platform (Illumina, San Diego, CA) at the National Microbiology Laboratory Canada. Data were analyzed using Excel and Stata, v15 (StataCorp, Texas).

## Results

The 23 participating acute care hospitals were located in six Canadian provinces: British Columbia (*n* = 7), Alberta (*n* = 2), Saskatchewan (*n* = 2), Ontario (*n* = 7), Quebec (*n* = 4), and New Brunswick (*n* = 1); the prospective cohort study for CPO colonization was taking place in south-central Ontario. During the study period, 488 at risk patients were screened: 45 in Group 1, 58 in Group 2, 103 in Group 3, 282 in Group 4, and none in Group 5. The median age of screened patients was 64 years (inter-quartile range (IQR) 42–74) and 253 (51.8%) were male.

No *C. auris* isolates were identified among 282 patients from populations that are normally associated with higher antifungal use (Group 4) in Canada during the study period. This group included patients with haematologic malignancies or solid organ transplants (*n* = 152 patients), and patients admitted to intensive care units (*n* = 109) or oncology wards (*n* = 21). The median age of patients in Group 4 was 63 years and 56.5% were male. In the 30 days prior to screening, 244 (89%) of these patients had received an antibiotic and 85 (38%) an antifungal; 167 (68%) had a central venous catheter. Five of these patients reported travel outside North America without healthcare in the previous 12 months (3 to the Caribbean, 2 to East Asia, 1 to the United Kingdom) and one patient received healthcare abroad (in Central Africa).

**Fig. 1:**
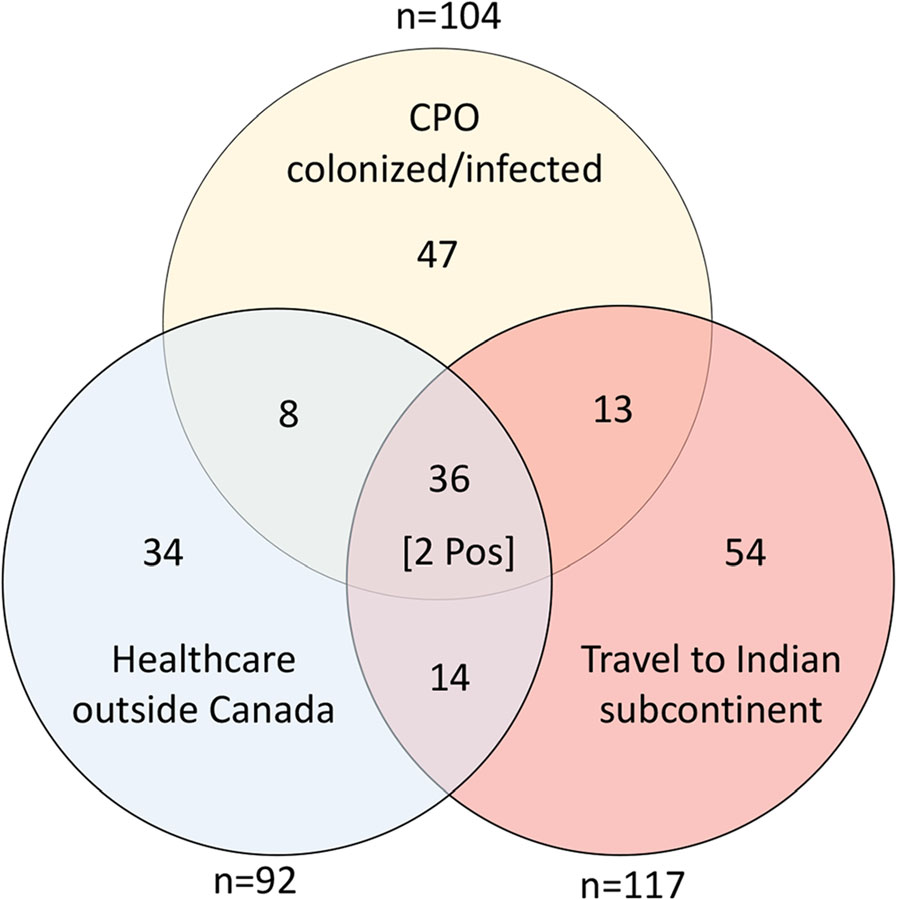
Overlapping risk factors in 206 patients who had recent hospitalization outside Canada, recent travel to the Indian subcontinent, or previous/current CPO colonization. The number of instances of each risk factor is shown outside of each circle. Healthcare outside of Canada was received in India (*n* = 37), the United States of America (*n* = 11), Pakistan (*n* = 6), China (*n* = 6), Portugal (*n* = 3), or other countries (*n* = 29). CPO colonized patients carried NDM (*n* = 59), OXA-48 (*n* = 24), both NDM and OXA-48 (*n* = 9), KPC (*n* = 11), or VIM (*n* = 1). One third of patients in these three groups had more than one risk factor (71 of 206 patients). Of 36 patients who had all three risk factors, 35 received healthcare in the Indian subcontinent and one received healthcare in the Netherlands. The two patients who were positive for *C. auris* had recently travelled to India and received healthcare there, and were both co-colonized with NDM-1 and OXA-48-like carbapenemase-producing organisms

There was considerable overlap in travel and healthcare associated risk factors in the 206 patients screened as part of Group 1 (hospitalization outside of Canada), Group 2 (travel to the Indian subcontinent without hospitalization) and Group 3 (CPO infection/colonization) (Fig. 1). The median age of 92 patients who had healthcare outside of Canada was 68 years and 58.7% were male. The median time between overseas healthcare admission and screening in Canada was 5.8 months (interquartile range 2.8–9.6). Healthcare was received in India (*n* = 37, 40%), the United States of America (*n* = 11, 12%), Pakistan (*n* = 6, 7%), China (*n* = 6, 7%), Portugal (*n* = 3, 3%), or other countries (*n* = 29, 32%). Among 117 patients who travelled to the Indian subcontinent without hospitalization, the median age was 63 years and 43.6% were male. Among 104 CPO colonized patients, the media age was 55 years and 51% were male. CPO colonized patients carried NDM (*n* = 59), OXA-48 (*n* = 24), both NDM and OXA-48 (*n* = 9), KPC (*n* = 11), or VIM (*n* = 1). Of 36 patients who had all three risk factors, 35 received healthcare in the Indian subcontinent and one received healthcare in the Netherlands. Two of these patients were found to be colonized with *C. auris*. *C. auris* was initially identified in both patients by MALDI-TOF and the results were confirmed by whole genome sequencing. Both *C. auris* colonized patients were hospitalized in India approximately 4 months prior to being screened for *C. auris* and both patients were known to be co-colonized with both NDM-1 and OXA-48-like producing organisms.

Thus, the overall prevalence of *C. auris* was 0.4% (2/488 patients; 95% CI 0.1–1.5%) in Canadian patients who were deemed to be at higher risk of fungal infections. Among the 35 patients in this study who had recently received healthcare in the Indian subcontinent and were CPO colonized or infected, the prevalence of *C. auris* colonization was 5.7% (95% CI 0.7–19.2%) compared to 0 of 453 without these two risk factors (*p* = 0.005).

One isolate was identified by both direct culture and broth pre-enrichment while the second isolate was identified only after a pre-enrichment for *C. auris* in broth. The second patient was undergoing daily chlorhexidine baths, which may have led to a lower burden of *C. auris*. A previous study found that direct culture resulted in detection of *C. auris* in 75% of the specimens that were positive by broth pre-enrichment [22]. At the time of *C. auris* detection, both patients were already under contact precautions due to their CPO colonization status; contact screening for *C. auris* was not carried out. Whole genome sequencing and phylogenetic analysis showed that both isolates belonged to South Asian Clade I, consistent with recent exposure to healthcare in India. In our study, the isolates were identified at the same healthcare facility, however they differed by 70 single nucleotide variants (SNVs), consistent with separate introductions into the facility. Within Clade 1, the US CDC previously found that genetically distinct isolates from multiple states differed by 62 SNVs (range 41–88) whereas isolates in an epidemiologically-linked cluster differed by a median of three SNVs (range 0–12) [3].

## Discussion

In this study, the overall prevalence of colonization with *C. auris* among Canadian patients at potential risk of colonization was low (0.4%). *C. auris* was only identified in patients who were both colonized/infected with CPO and who had recently received inpatient healthcare in the Indian subcontinent.

Our findings support the hypothesis that the greatest risk from *C. auris* is posed by spread of existing clades from endemic areas to new areas, as opposed to the emergence of new strains associated with increasing antifungal use. Importation of all four clades of *C. auris* into the United States from other countries has also been documented [3]. These data are consistent with the recommendation of the US CDC for admission screening of patients who have had an overnight hospitalization in the previous 12 months in a country where *C. auris* has been documented, especially if they also have a CPO [23]. However, our study did not evaluate all risk groups. For instance, our study did not screen residents of long term acute care facilities, who have been found to be a risk group in the United States [24]. Although *C. auris* colonization currently appears to be uncommon in Canadian hospitals, its rapid spread in other countries suggests that Canadian hospitals should consider active screening of high risk groups and contacts of index cases to permit early detection and limit the spread of *C. auris* [5].

## Data Availability

Sequence reads were deposited into the National Centre for Biotechnology Sequence Read Archive (Biosamples SAMN13424501 and SAMN13424502 in Bioproject PRJNA592373).
